# Primary Renal Mesenchymal Tumors: A Study from North India, Emphasizing Rare Entities and Molecular Profiles

**DOI:** 10.15586/jkc.v13i3.505

**Published:** 2026-09-17

**Authors:** Alka Yadav, Pallavi Prasad, Ankita Singh, Vinita Agrawal, Uday Pratap Singh

**Affiliations:** 1 Department of Pathology, Sanjay Gandhi Postgraduate Institute of Medical Sciences (SGPGIMS), Lucknow, Uttar Pradesh, India;; 2 Department of Urology and Renal Transplantation, Sanjay Gandhi Postgraduate Institute of Medical Sciences (SGPGIMS), Lucknow, Uttar Pradesh, India

**Keywords:** angiomyolipoma, benign, histopathology, mesenchymal, nephrectomy, primary renal mesenchymal tumors

## Abstract

Primary renal mesenchymal tumors (PRMT) are uncommon, heterogeneous, and diagnostically challenging neoplasms. Comprehensive institutional data on the prevalence, clinicopathological features, immunohistochemical and molecular profiling, and outcomes of PRMTs in South Asia are limited. This retrospective study analyzed 64 surgically resected PRMTs from 1975 nephrectomies performed at our institution between January 2014 and June 2024. Tumors were categorized according to the World Health Organization (WHO) 2022 classification system. Clinico-radiological and histopathological, treatment and follow-up data were retrieved from the hospital information systems. Among all surgical nephrectomies, 64 PRMTs were identified (prevalence 3.2%). According to the WHO 2022 classification, 37 tumors (58%) were benign, 22 (34%) were malignant, and 5 tumors (8%) were intermediate. The overall female-to-male ratio was 1.1:1. Adults comprised 81.2% of the patients (n = 52), children comprised 18.8% (n = 12). Angiomyolipoma was the most common tumor, comprising 56% of PRMTs (n = 36), followed by clear cell sarcoma of the kidney (n = 7; 11%), Ewing sarcoma (n = 6; 9%), leiomyosarcoma (n = 6; 9%), congenital mesoblastic nephroma (n = 3; 5%), and other rare entities. The mean follow-up duration was 41.6 months, with recurrence in three cases and metastasis in six cases. This is one of the largest Indian series conducted on PRMTs, documenting a diverse spectrum. Malignant tumors tend to occur at a younger age, although exceptions have been reported. The occurrence of extremely rare entities highlights the critical role of immunohistochemistry and molecular testing in the accurate classification and treatment planning of these tumors.

## Introduction

Primary renal mesenchymal tumors (PRMTs) are rare and heterogeneous, accounting for approximately 1–2% of resected renal masses (1). Their behavior ranges from benign to malignant sarcomas. They may present as primary renal masses or incidental tumors during nephrectomies for renal or urothelial malignancies or an end-stage renal disease. Accurate classification, supported by immunohistochemistry (IHC) and molecular testing, is crucial because management, prognosis, and eligibility for targeted therapies vary across entities, resulting in diagnostic challenges for pathologists.

The World Health Organization (WHO) Classification of Tumors of the Urinary System and Male Genital Organs, 2022 (5th edition) provides a taxonomy that has molecular integration (2), categorizing these tumors based on biological behavior into benign, intermediate, and malignant entities (Table 1). However, South Asian series applying this classification to surgical nephrectomy cohorts are absent. Most PRMT series involve Western populations, including the 16-year survey done by Alruwaii et al. (3); their surgical/incidental or autopsy-based methodology differs from this nephrectomy cohort. A clinicopathological study of 31 surgically resected renal angiomyolipomas (AMLs) from the institution (4) provided foundation for the PRMT spectrum documented here. This 10-year study of 64 PRMTs from SGPGIMS, Lucknow, India, emphasized the WHO 2022 behavior classification (2) with pediatric/adult stratification, AML subtype-specific age distribution, IHC, including surrogate molecular markers, recurrence, metastasis, and rare entities, including adult clear cell sarcoma of the kidney (CCSK), adult malignant rhabdoid tumor (MRT), inflammatory myofibroblastic tumor (IMT), and primary renal angioleiomyoma. This is one of the largest PRMT nephrectomy series, aiding in understanding the differential diagnosis and treatment options.

**Table 1:** World Health Organization classification of primary renal mesenchymal tumors.

Tumors occurring mainly in adults	Tumors occurring mainly in children
Leiomyosarcoma	Clear cell sarcoma
Angiosarcoma	Congenital mesoblastic
Rhabdomyosarcoma	nephroma
Ewing sarcoma	Rhabdoid tumor
Synovial sarcoma	Ossifying renal tumor of
Osteosarcoma	infancy
Angiomyolipoma	
Epithelioid	
angiomyolipoma	
Solitary fibrous tumor	
Leiomyoma	
Hemangioma	
Lymphangioma	
Hemangioblastoma	
Juxtaglomerular cell tumor	
Renomedullary interstitial cell tumor	

## Material and Methods

This retrospective descriptive observational study included resected nephrectomies with a primary histopathological diagnosis of mesenchymal tumors at SGPGIMS, Lucknow, India, from January 2014 to June 2024. The study was conducted in accordance with the ethical principles of the Declaration of Helsinki (2013, revision). PRMTs with adequate clinicopathological data were included. Metastatic or retroperitoneal sarcomas extending to the kidney, vena cava or renal vein tumors, mixed epithelial-mesenchymal tumors, needle biopsies without IHC-confirmed diagnosis, and patients with insufficient data were excluded. Hospital information system (HIS) provided clinico-radiological, pathological, treatment, and follow-up data. Tumors were classified according to WHO 2022 classification (2) by behavior and age group. IHC on formalin-fixed paraffin-embedded (FFPE) sections used the Ventana BenchMark platform. IHC served as a surrogate molecular marker for anaplastic lymphoma kinase (ALK) in IMT, integrase interactor 1 (INI1/SMARCB1) in MRT, and *SS18::SSX* fusion in synovial sarcomas. Data were analyzed using IBM SPSS Statistics version 25.0 (IBM Corp., Armonk, NY, USA). Categorical variables were expressed as frequencies and percentage values, and continuous variables were expressed as mean ± standard deviation (SD) or range.

## Results

A total of 1975 nephrectomies were performed over 10 years, of which 64 (3.2%) were for PRMTs. The cohort comprised 34 females and 30 males, with a female-to-male ratio of 1.1:1 and ages ranging from 1 month to 83 years (mean age: 41.0 ± 35.9 years). Right kidney involvement was noted in 35 (55%) patients and left kidney involvement in 29 (45%). A brief outline is provided as follows: The mean tumor dimension was 10.6 cm (range: 1.0–46.0 cm). According to the WHO 2022 biological behavior classification (2), 37 tumors were benign (58%), five intermediate (8%), and 22 malignant (34%). In all, 52 were adult patients (81%) and 12 were pediatric patients (19%). Complete distribution based on the diagnosis, WHO 2022 biological behavior, and adult/pediatric stratification is summarized in Table 2.

**Table 2:** Distribution of primary renal mesenchymal tumors by WHO 2022 behavior (2) and adult/pediatric stratification (n = 64).

Tumor	WHO 2022 behavior (2)	n (%)	Adult (≥18 years)	Pediatric (<18 years)	M–F ratio	Mean size (range) (cm)	Mean age (range) (years)	Site R:L
**Adult: benign**								
Classical AML	Benign	28 (44%)	28	-	8:20	10.4 (2.5–20.5)	51.2 (28–80)	16:12
Leiomyoma-like AML	Benign	5 (8%)	5	-	2:3	4.4 (3.0–5.8)	44.6 (35–57)	3:2
Lipoma-like AML	Benign	1 (2%)	1	-	0:1	11.0	37	1:0
Angioleiomyoma	Benign	1 (2%)	1	-	1:0	1.5	56	1:0
**Adult: intermediate**								
Epithelioid AML*	Intermediate	1 (2%)	1	-	0:1	10.0	40	1:0
IMT	Intermediate	1 (2%)	1	-	1:0	3.5	40	0:1
**Adult: malignant**								
LMS	Malignant	6 (9%)	6	-	4:2	14.3 (8.0–17.0)	68.8 (54.0–83.0)	1:5
EWS	Malignant	4 (6%)	4	-	2:2	12.8 (6.5–12.7)	32.3 (20.0–40.0)	3:1
CCSK	Malignant	2 (3%)	2	-	2:0	15.0 (10.0–20.0)	27.0 (20.0–34.0)	2:0
MRT	Malignant	1 (2%)	1	-	0:1	8.0	28.0	1:0
Synovial sarcoma	Malignant	1 (2%)	1	-	1:0	4.6	51.0	0:1
Mesenchymal chondrosarcoma	Malignant	1 (2%)	1	-	1:0	8.0	32.0	1:0
**Pediatric: benign**								
Lipoma‡	Benign	1 (2%)	-	1	1:0	1.0	1.0	0:1
Leiomyoma	Benign	1 (2%)	-	1	1:0	3.7	9.0	0:1
**Pediatric: intermediate**								
Epithelioid AML*	Intermediate	1 (2%)	-	1	0:1	10.0	6.0	0:1
CMN	Intermediate§	2 (3%)	-	2	1:1	9.0 (6.0–12.0)	1.6 (0.3–3.0)	1:1
**Pediatric: malignant**								
CCSK	Malignant	5 (8%)	-	5	4:1	10.3	8.0 (3.0–13.0)	3:2
EWS	Malignant	2 (3%)	-	2	1:1	20.8 (17.4–25.0)	14.5 (14.0–15.0)	1:1
**Total**	**-**	**64 (100%)**	**52 (81%)**	**12 (19%)**	**30:34**	**10.6 (1.0–25.0)**	**41.0 ± 35.9 (0.3–83.0)**	**35:29**

Notes. Bold rows = WHO 2022 behavior category headings (not separate entities).

* Epithelioid AML (EAML): total 2: adult 1 (40 years, F) and pediatric 1 (6 years, F), listed separately.

‡ Pediatric lipoma: melanocytic markers not performed; categorized morphologically.

§ CMN: intermediate overall according to the WHO 2022 behavior classification; the classic variant behaves effectively benign (EGFR-ITD); the cellular variant has ~10% recurrence risk (*ETV6::NTRK3* fusion).

AML: angiomyolipoma; IMT: inflammatory myofibroblastic tumor; LMS: leiomyosarcoma; EWS: Ewing sarcoma; CCSK: clear cell sarcoma of the kidney; MRT: malignant rhabdoid tumor; CMN: congenital mesoblastic nephroma; EGFR-ITD: epidermal growth factor receptor internal tandem duplication.

### Adult benign tumors

*Classical AML* (n = 28) was the predominant tumor, comprising all adult cases (mean age: 51.2 years, range: 28–80 years) with female predilection (F–M ratio of 2.5:1). The mean tumor dimension was 10.4 cm (range: 2.5–20.5 cm), and tuberous sclerosis complex (TSC) was associated in three cases. Detailed clinicopathological data from 31 AML patients (including 24/28 classical AML patients) at our institution were published separately (4). *Leiomyoma-like AML* (LMAML; n = 5, all adults, mean age: 44.6 years) had a mean tumor size of 4.4 cm; one case had concurrent ipsilateral clear-cell renal cell carcinoma (RCC). *Lipoma-like AML* (LLAML; n = 1, 37-year-old female, tumor size: 11.0 cm) was distinguished from lipoma by human melanoma black-45 positivity (HMB-45+), classifying it as a fat-predominant AML, with implications of TSC surveillance (5). In three of the five LMAML patients lacking triphasic morphology, HMB-45 was positive in all three and smooth muscle actin (SMA) in two patients (4).

**Figure 1:**
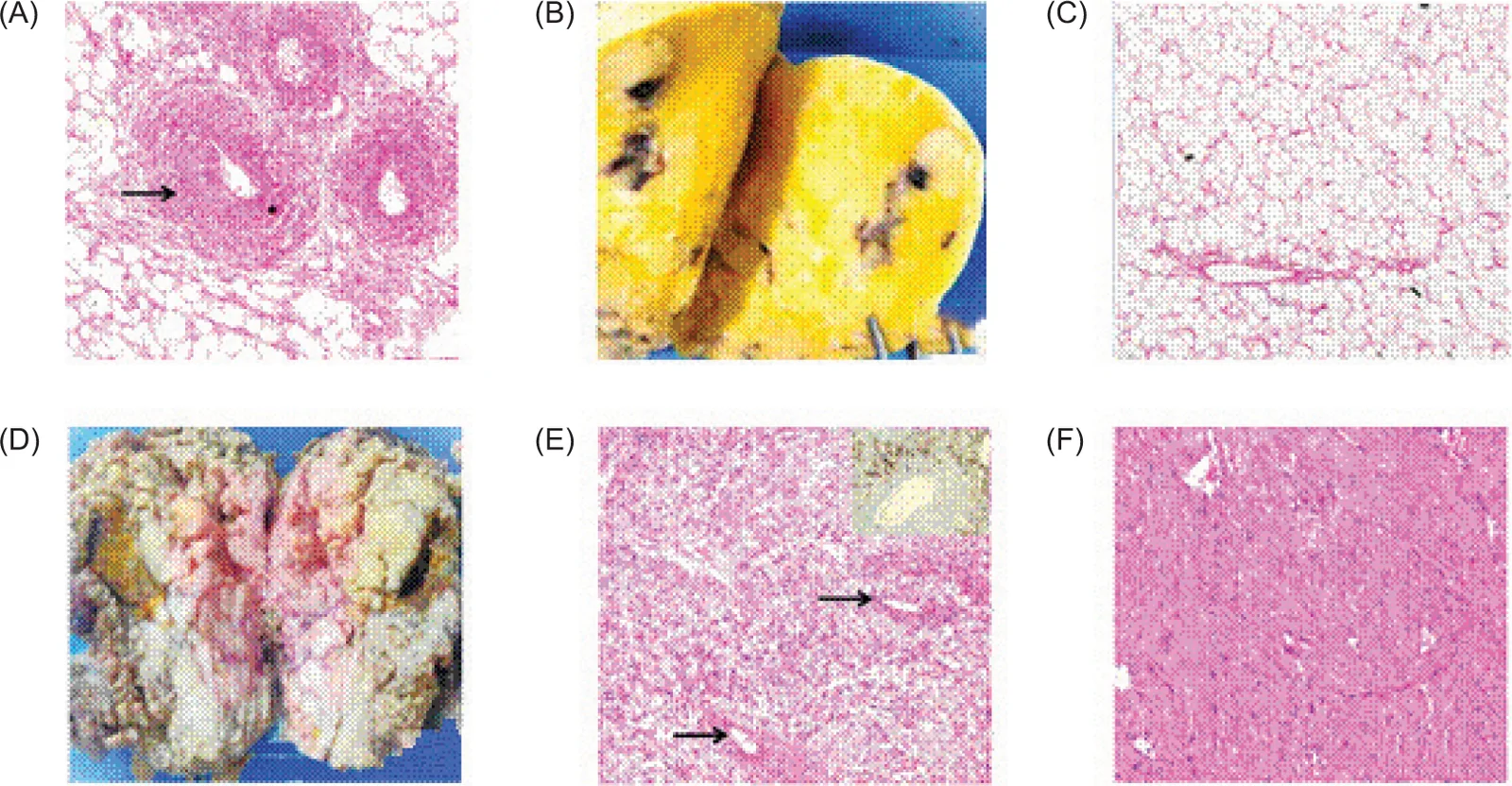
Classical angiomyolipoma: (A) histology shows malformed blood vessels (black arrows), muscle, and fat. Lipoma-like angiomyolipoma: (B) renal mass with predominantly fatty cut surface; (C) histology resembles lipoma, with occasional blood vessels (hematoxylin and eosin staining [H&E], ×100). Leiomyoma-like angiomyolipoma: (D) gray-white renal mass with lobulated fat and hemorrhage; (E) tumor composed of spindle-shaped cells and thick-walled blood vessels (black arrows) (H&E, ×100); HMB-45 immunostain positive (IHC, ×100; inset). Angioleiomyoma: (F) histology shows smooth muscle bundles surrounding thick-walled blood vessels (H&E, ×100).

*Primary renal angioleiomyoma* (n = 1) occurred in a 56-year-old male with a 1.5-cm right renal mass; IHC showed SMA positivity, focal desmin/vimentin positivity, CD34/HMB-45/pan-cytokeratin (Pan-CK)/S100 negativity, and protein Ki-67 proliferative index <1%. This entity is rare, with fewer than 10 cases reported in the literature till date (6). HMB-45 negativity (HMB-45-) is the key discriminator of LMAML (5). The histopathological features are illustrated in Figure 1.

### Adult intermediate-behavior tumors

*Epithelioid AML* (EAML)—adult (n = 1): A 40-year-old female with a 10.0-cm renal mass underwent radical nephrectomy. Histopathology revealed polygonal cells with round nuclei and eosinophilic cytoplasm. HMB-45 and SMA were IHC-positive. Tumor deposits involved hilar and para-aortic lymph nodes. Brimo et al.’s criteria suggestive of aggressive EAML features were absent (7). At follow-up, she was alive without progression, suggesting multicentric perivascular epithelioid cell tumor (PEComa) rather than true metastasis (see “Recurrence and Metastasis” section).

**Figure 2:**
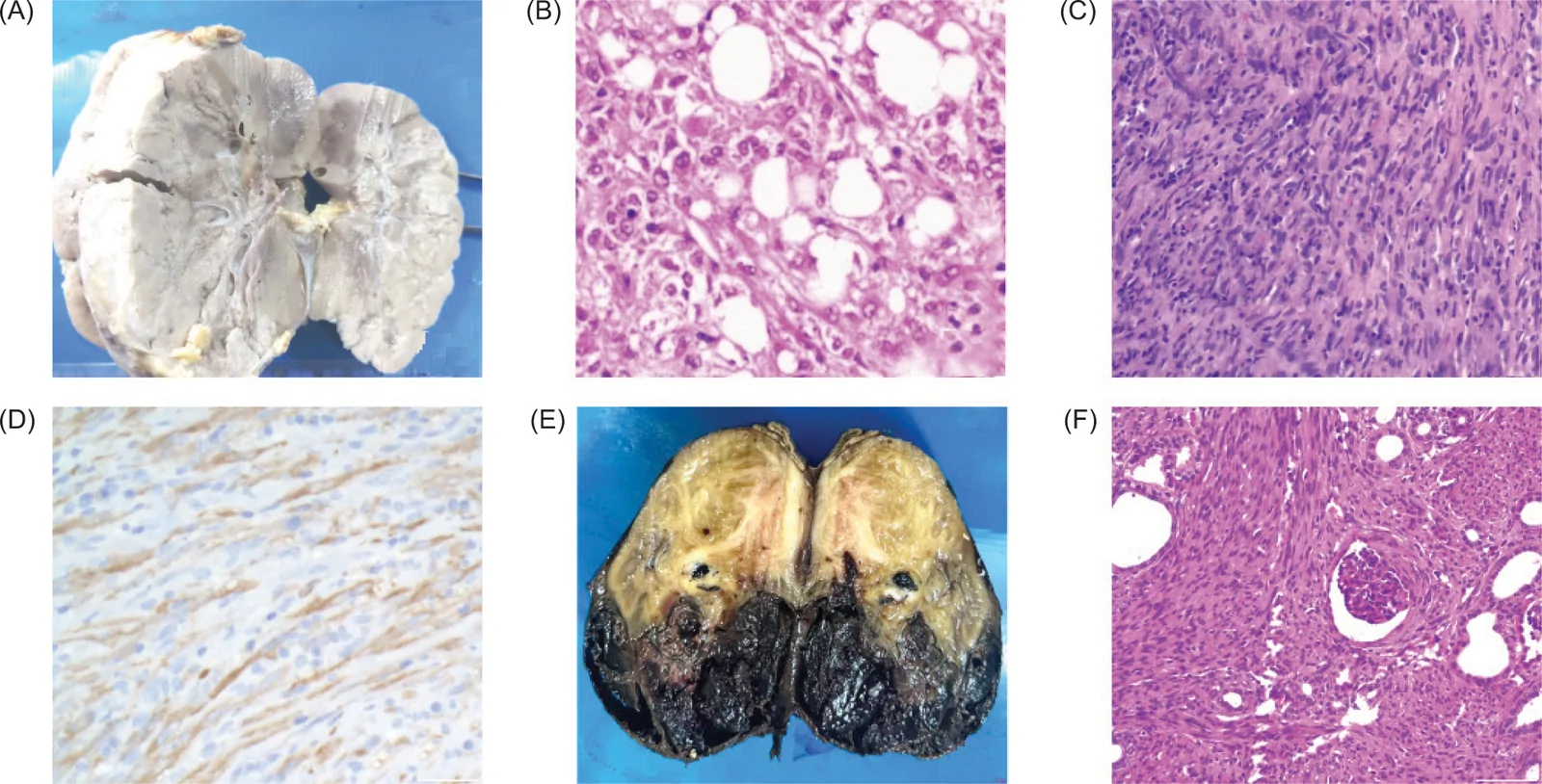
Epithelioid angiomyolipoma: (A) gross image showing a gray**-**tan, firm growth replacing the entire renal parenchyma; (B) photomicrograph showing polygonal to spindle-shaped tumor cells with round-to-oval nuclei and eosinophilic cytoplasm arranged in nests or sheets (hematoxylin and eosin staining [H&E], ×400). Inflammatory myofibroblastic tumor: (C) histology shows spindle cells in a collagenous background with lymphocytes and plasma cells (H&E, ×200); (D) ALK immunohistochemistry, clone ALK1 (CD246), shows diffused cytoplasmic staining in tumor cells (H&E, ×400). Congenital mesoblastic nephroma: (E) gross image showing a gray-white whorled mass occupying the kidney; (F) histology showing fascicles of spindle-shaped cells with entrapped renal elements (H&E, ×200).

*Inflammatory myofibroblastic tumor* (n = 1): A 40-year-old male with a 3.5-cm renal mass underwent partial nephrectomy. Histopathology revealed myofibroblastic spindle cells with lymphoplasmacytic infiltrate disposed in myxoid-fibrous stroma. IHC revealed SMA, vimentin, and CD68 positivity, and negativity for CD34, CD10, PAX8, and desmin. ALK IHC (clone ALK1/CD246 antibody) was positive, confirming IMT. Although ~50% of IMTs are ALK-negative by IHC, IMT is not excluded; fluorescence *in situ* hybridization (FISH) or RNA-based fusion testing can detect rearrangements and identify actionable targets (8). No recurrence or metastasis was observed. The histopathological and immunohistochemical features are presented in Figure 2.

### Adult malignant tumors

*Leiomyosarcoma* (LMS; n = 6): average age 68.8 years (range: 54–83 years), male predominance (M–F ratio of 2:1). Mean tumor dimension 14.3 cm (range: 8–17 cm). Histopathology showed spindle-cell fascicles, eosinophilic cytoplasm, blunt-ended nuclei, and 5/10–40/10 high-power field (HPF) mitoses. Necrosis occurred in four (67%) patients. SMA was positive (5/5); 4/5 patients were desmin-positive and Pan-CK/myogenin-negative. Ki-67 index ranged from 5 to 35%. One patient showed inferior vena cava (IVC) infiltration, one had transverse colon infiltration, and one lung metastasis; two patients showed recurrent renal LMS.

*Ewing sarcoma* (EWS) in adults (n = 4): age was 20, 31, 38, and 40 years; M–F ratio of 1:1. The mean tumor size was 12.8 cm. CD99 positivity was observed in 3/4 tested cases; one CD99-negative case expressed synaptophysin, CD56, and neuron-specific enolase (NSE) was classified as EWS according to the WHO 2022 classification (2). A post-chemotherapy nephrectomy revealed a residual Wilms tumor, initially misdiagnosed as EWS on morphology and CD99 positivity, highlighting the misclassification risk without EWSR1 FISH.

*Clear cell sarcoma of the kidney*—adult (n = 2): Aged 20 and 34 years, both males, with tumor size of 20 cm and 10 cm, respectively; both patients underwent radical nephrectomy. Histopathology revealed classic oval-to-spindle-shaped cells in a fibromyxoid stroma with branching capillaries. IHC showed CCND1 (cyclin D1) nuclear positivity, BCL2 cytoplasmic positivity, focal vimentin positivity, and negative WT1, synaptophysin, SMA, chromogranin, and keratin. In the 20-year-old patient, biopsy-confirmed abdominal wall locoregional recurrence was proved fatal; the 34-year-old patient was disease-free at follow-up.

*Malignant rhabdoid tumor* (n = 1): A 28-year-old female presented with a right renal mass, flank pain, and gross hematuria and underwent nephroureterectomy. Histopathology showed discohesive cells with eccentric nuclei, prominent nucleoli, hyaline intracytoplasmic inclusions, necrosis, and 18–20 mitotic figures/10 HPFs. IHC showed vimentin, SMA, CK, CD99, and S100 positivity, and CD34, CK7, CK20, desmin, myogenin, HMB-45, leukocyte common antigen (LCA), PAX8, GATA-3, BCL2, and β-catenin negativity. INI1 (SMARCB1) showed complete loss of nuclear expression in tumor cells, the hallmark IHC marker according to the WHO 2022 classification (2).

*Synovial sarcoma* (SS; n = 1): A 51-year-old male presented with metastasis in the right femoral neck at the diagnosis, and a left radical nephrectomy confirmed primary tumor. Histopathological examination of a 4.6-cm left renal mass revealed monophasic spindle cells with hemangiopericytoma-like blood vessels. IHC showed vimentin and focal CK7 positivity, and ALK, CD10, CK20, and AMACR negativity. *SS18::SSX* IHC revealed nuclear positivity. The Ki-67 index was approximately 30–40%.

**Figure 3:**
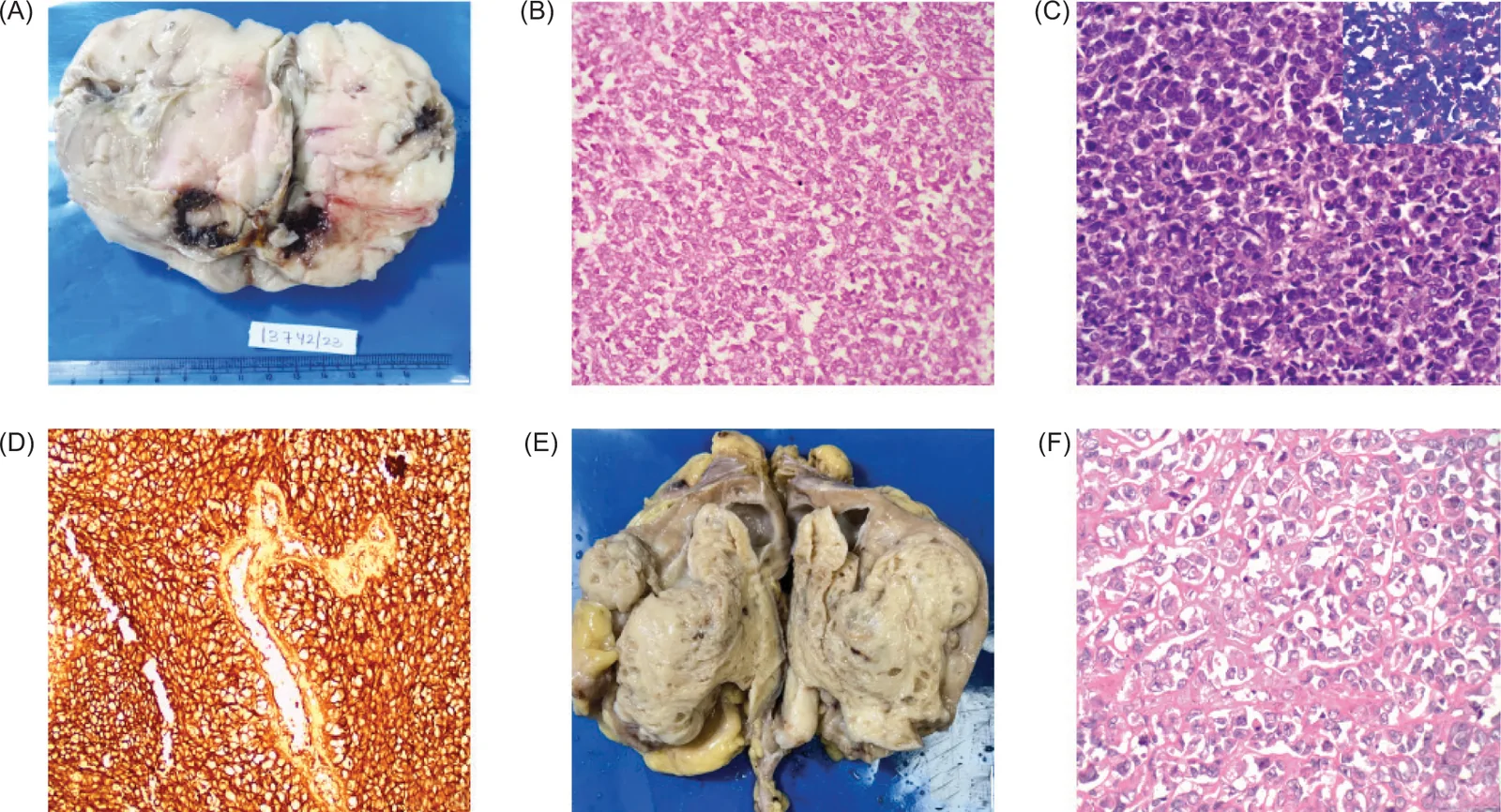
Clear cell sarcoma: (A) gross specimen showing a smooth, gray**-**white, whorled mass occupying most of the kidney; (B) histology with monomorphic round blue cells (hematoxylin and eosin staining [H&E], ×200). Ewing sarcoma: (C) histopathology with sheets of small round blue cells, scant cytoplasm, hyperchromatic nuclei, and indiscernible borders (H&E, ×400); tumor cells show strong cytoplasmic positivity for PAS stain, consistent with intracytoplasmic glycogen (PAS, ×400, inset); (D) immunohistochemistry revealed diffused, strong membranous CD99 positivity, supporting the diagnosis (H&E, ×400). Malignant rhabdoid tumor: (E) gross images of a lobulated, fleshy, gray-white mass with irregular margins; (F) histology displays non-cohesive cells with large, eccentric nuclei, prominent nucleoli, and intracytoplasmic inclusions (H&E, ×400).

**Figure 4:**
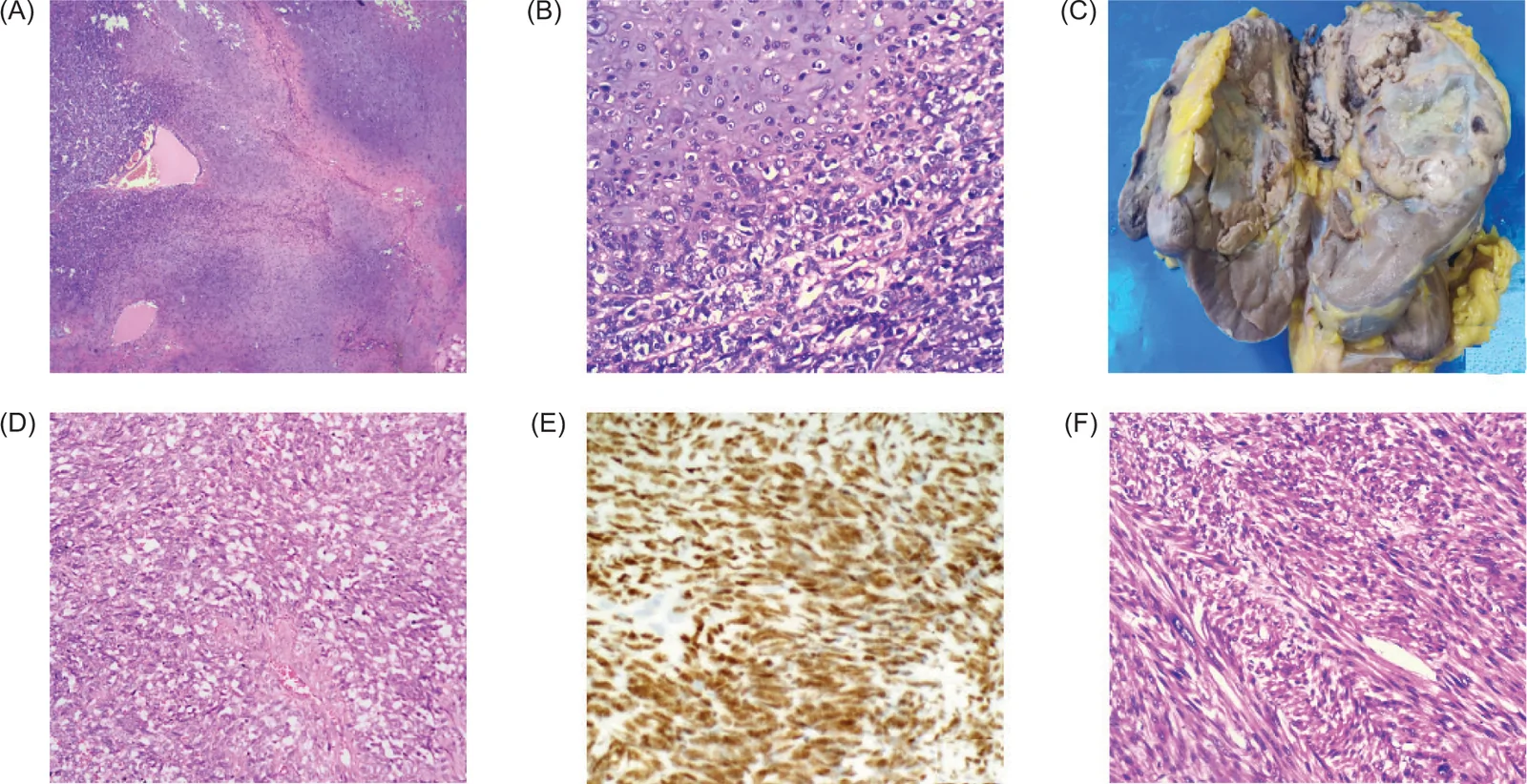
Mesenchymal chondrosarcoma: (A) low-power view of a biphasic tumor with hypocellular cartilaginous islands and hypercellular areas of small round cells with ectatic thin-walled vascular channels, adjacent to well-differentiated cartilage (hematoxylin and eosin staining [H&E], ×40); (B) higher magnification of islands of well-differentiated cartilage (upper-left) and undifferentiated small round-to-spindle cell sheets (lower-right) with hyperchromatic nuclei (H&E, ×400). Synovial sarcoma: (C) nephrectomy specimen with fleshy, gray**-**white, necrotic mass; (D) histology shows a cellular neoplasm of monomorphic spindled cells in a fascicular pattern and thin-walled vessels with compressed lumen (H&E, ×200); (E) immunohistochemistry with diffused nuclear *SSX::SS18* expression in tumor cells (H&E, ×400). Leiomyosarcoma: (f) histology shows intersecting spindle-cell fascicles with eosinophilic cytoplasm, cigar-shaped nuclei with blunt ends (H&E, ×200).

*Mesenchymal chondrosarcoma* (MCS; n = 1): A 32-year-old male presented with a right renal mass and underwent radical nephrectomy. The dimorphic pattern comprising undifferentiated small round cells alternating with hyaline cartilage confirmed the diagnosis. No recurrence or metastasis was observed. Representative gross, histopathological, and IHC features of major malignant tumors are shown in Figures 3 and 4.

### Pediatric benign tumors

*Lipoma* (n = 1): A 1-year-old boy presented with an incidental 1.0-cm left renal mass detected during robotic pyeloplasty for pelvi-ureteric junction obstruction. Histomorphology showed mature adipose tissue without atypia or lipoblasts. IHC with melanocytic markers (HMB-45, Melan-A, and cathepsin K) was not performed, a limitation, as even focal positive expression would reclassify the lesion as fat-predominant AML (5).

*Leiomyoma* (n = 1): A 9-year-old boy presented with left upper abdominal pain. Imaging revealed a 3.7-cm enhancing left renal mass; cystic RCC and LMS were considered—laparoscopic partial nephrectomy was performed. The tumor was gray-white and developed from right renal capsule. Histopathology showed intersecting fascicles of bland spindle cells without atypia, mitosis, or necrosis. IHC showed vimentin, desmin, and CD34 positivity, and pan-cytokeratin and S100 negativity; and a Ki-67 index < 1% confirmed primary renal leiomyoma. Postoperative course was uneventful; disease-free at 1-year follow-up.

### Pediatric intermediate-behavior tumors

*Epithelioid AML* (n = 1): A 6-year-old girl presented with a 10-cm right renal mass and underwent radical nephrectomy. Tumor deposits were identified in the hilar and para-aortic lymph nodes. On IHC, HMB-45 and SMA showed positive expression, whereas PAX8 and Pan-CK were negative. Similar to the adult EAML case, histological criteria suggestive of aggressive EAML were not met, and lymph node deposits were interpreted as multifocal PEComa (see “Recurrence and Metastasis” section).

*Congenital mesoblastic nephroma* (CMN; n = 2, both pediatric): The patients presented at 3 months and 3 years of age. According to the WHO 2022 classification, CMN shows intermediate behavior (2, 9). Subtype-specific interpretation: the classic variant, driven by epidermal growth factor receptor (EGFR) internal tandem duplication (EGFR-ITD), behaves benignly, and is cured by total nephrectomy, with a recurrence rate of <2% (9). Cellular variant, driven by *ETV6::NTRK3* gene fusion, is analogous to infantile fibrosarcoma, with a 10% recurrence risk, mostly with renal sinus invasion or positive resection margins. Relapses were reported almost exclusively within 12 months post-surgery (10). One cellular CMN (in a 3-year-old boy, 6-cm left renal mass) had renal sinus involvement and 6–8 mitotic figures/10 HPF, a criterion for heightened postoperative surveillance. One classic CMN had favorable histological features. Molecular testing was unavailable (11). Neither of the patients developed recurrence or metastasis.

### Pediatric malignant tumors

*Clear cell sarcoma of the kidney*, pediatric (n = 5): Age at presentation was 3, 5, 6, 13, and 13 years, with a M–F ratio of 4:1. The mean tumor size was 10.9 cm (range: 8–12 cm). All patients underwent radical nephrectomy. Histopathological examination revealed a classic pattern in each case. IHC confirmed CCND1 nuclear and BCL2 cytoplasmic positivity in all cases. Wilms Tumor 1 (WT1), synaptophysin, and SMA were also positive. One 3-year-old patient had pN1 disease with retrocaval and preaortic lymph node involvement, and another 5-year-old had interaortocaval lymph node involvement, a regional metastasis. A 13-year-old patient had tumor thrombus in the renal vein and IVC with isolated lung metastasis. At follow-up, three were alive; however two patients were lost to follow-up.

*Ewing sarcoma*, pediatric (n = 2): At presentation, patients were aged 14 and 15 years, with a M–F ratio of 1:1. The mean tumor size was 20.8 cm. CD99 (diffused membranous), vimentin, and NSE positivity, along with supportive periodic acid–Schiff (PAS) staining, were confirmed in both cases. Results for WT1, Pan-CK, LCA, and desmin were negative. No metastasis or recurrence was documented.

### Locoregional recurrence, distant metastasis, and lymph node tumor deposits

Locoregional recurrence occurred in three patients (4.7%): two LMS cases (retroperitoneum and renal fossa) and one adult CCSK (abdominal wall). Metastasis occurred in six patients (9.4%): one LMS case (lung involvement), one SS case (right femoral neck), three pediatric CCSK cases (pN1 retrocaval/preaortic nodes, 3 years ×1; pN1 interaortocaval node, 5 years ×1; renal vein and IVC thrombus with lung metastasis, 13 years ×1), and one synchronous epithelial biliary tract malignancy with classical AML with metastatic deposits at station 13 and cystic lymph nodes confirmed by Pan-CK (deceased at last follow-up). Tumor behavior, staging, and molecular characteristics of all histological types are summarized in the Table 3 (12–14). Table 4 summarizes the outcomes of non-AML tumors.

**Table 3:** Clinicopathological, staging, and molecular characteristics of primary renal mesenchymal tumors.

Histology; n (%)	Median age (range)	Stage (COG/AJCC TNM)	Extent of disease	Molecular alteration/mutation status
**Benign tumors**				
Classical AML; 28 (44%)	53.5 (28–80)	Not clinically relevant (2)	Kidney-confined; regional nodal deposits in 1/28 cases (cystic and station 13 lymph nodes) of non-PRMT/biliary tract epithelial malignancy	Not tested; 2 TSC associated cases§
Leiomyoma-like AML; 5 (8%)	39 (35–57)	Not clinically relevant (2)	Kidney-confined	Not tested
Lipoma-like AML; 1 (2%)	37	Not clinically relevant (2)	Kidney-confined	Not tested
Angioleiomyoma; 1 (2%)	56	Not clinically relevant (2)	Kidney-confined	Not tested
Lipoma; 1 (2%)	1	Not clinically relevant (2)	Kidney-confined	Not tested for *PTEN* sequencing
Leiomyoma; 1 (2%)	9	Not clinically relevant (2)	Kidney-confined	None tested
**Intermediate-behavior tumors**				
Epithelioid AML; 2 (3%)	23 (6–40)	Not applicable (2)	Regional nodal deposits in both the cases (6 years and 40 years); regarded as multifocal PEComa growth rather than true metastasis	Not tested; 1 TSC-associated case§
IMT; 1 (2%)	40	Not currently available, because the tumors usually behave favorably irrespective of their size (2)	Kidney-confined	ALK IHC positive (surrogate); FISH/NGS not performed
CMN; 2 (3%)	1.7 (0.3–3)	Stage II (0.3 years), stage II (3 years) (COG staging system),† completely resected	Renal sinus extension in both the cases (0.3 years and 3 years)	None tested; classic subtyped on morphology (literature reported EGFR-ITD) and cellular subtyped on morphology (ETV6::NTRK3 is a literature-associated fusion (2))
**Malignant tumors**				
LMS; 6 (9%)	72.0 (54.0–83.0)	All adult. pT1N0M0 ×2; pT2N0M0 ×1 (IVC wall invasion); pT1N0M0 ×1 (retroperitoneal recurrence during follow-up); pT3N0M0 ×1 (transverse colon invasion and recurrence during follow-up); pT1N0M1×1 (pulmonary metastasis). (AJCC TNM staging system)*	Invasion of IVC wall 1/6; invasion of transverse colon (sigmoidectomy performed) 1/6	None tested
EWS; 6 (9%)	25.5 (14.0–40.0)	Adult (n = 4): pT2N0M0 × 3 (renal sinus fat involvement in 2 and upper ureteric extension in 1); pT1N0M0 × 1 (no local extension, nodal or distant metastasis) (AJCC TNM staging system)*; pediatric (n = 2): COG stage I ×1; Stage II ×1 (perirenal fat and IVC tumor thrombus). All 6 cases were localized.	Renal sinus (3/6)/perirenal fat involvement 1/6; IVC tumor thrombus with vessel wall infiltration and lymphovascular invasion 1/6	EWSR1 rearrangement was not performed (CD99 with PAS stain was performed)
CCSK; 7 (11%)	13.0 (3.0–34.0)	Pediatric (n = 5): COG Stage I ×2 (13 years and 6 years, both kidney-confined); Stage III ×2 (3 years and 5 years, regional nodal deposits); Stage IV ×1 (13 years, pulmonary metastasis with renal vein and IVC tumor thrombi); pT2N0M0 (20 years, renal sinus fat involvement with subsequent abdominal wall recurrence); pT1N0M0 (34 years, kidney-confined) (AJCC TNM staging system)*	Perinephric fat involvement 1/7; renal vein plus IVC tumor thrombi 1/7; regional lymph node involvement 2/7 (retrocaval and pre-aortic; interaortocaval)	BCOR-ITD/ YWHAE::NUTM2 were not tested; CCND1/BCL2 IHC surrogate markers were performed
MRT; 1 (2%)	*28*	pT3N0M0 (AJCC TNM staging system)*	*Primary renal tumor with contiguous extension into renal pelvis and upper ureter; no regional lymph-node or distant metastasis*	*INI1 (SMARCB1) complete loss was confirmed on IHC*
SS; 1 (2%)	*51*	pT1N0M1 (right femoral neck metastasis) (AJCC TNM staging system)*	*Kidney-confined primary*	SS18::SSX FISH not performed; SS18::SSX IHC surrogate marker was performed
MCS; 1 (2%)	*32*	pT1N0M0 (AJCC TNM staging system)*	*Kidney-confined primary*	*None tested*

§ TSC-association based on clinical diagnostic criteria (12).

† Pediatric renal tumors were staged according to Children’s Oncology Group (COG)/National Wilms Tumor Study Group (NWTSG) staging system (13).

*AJCC TNM staging, 8th edition was applied descriptively using the visceral-organ soft-tissue sarcoma framework. Currently, no existing validated tumor-specific staging system (14).

AML: angiomyolipoma; AJCC: American Joint Committee on Cancer; ALK: anaplastic lymphoma kinase; BCOR: BCL6 corepressor; BCL2: B-cell lymphoma 2; COG: Children’s Oncology Group; CCSK: clear cell sarcoma of the kidney; CCND1: cyclin D1; CMN: congenital mesoblastic nephroma; EWS: Ewing sarcoma; EGFR: epidermal growth factor receptor; ETV6: ETS variant transcription factor 6; FISH: fluorescence *in situ* hybridization; IHC: immunohistochemistry; IMT: inflammatory myofibroblastic tumor; INI1: integrase interactor 1; ITD: internal tandem duplication; LMS: leiomyosarcoma; MCS: mesenchymal chondrosarcoma; MRT: malignant rhabdoid tumor; NGS: next-generation sequencing; NUTM2: NUT family member 2; PAS: periodic acid–Schiff; SS: synovial sarcoma; *SS18* (formerly *SYT*) gene; SMARCB1: SWI/SNF-related, matrix-associated, actin-dependent regulator of chromatin, subfamily B, member 1; TNM: tumor-node-metastasis; TSC: tuberous sclerosis complex; WHO: World Health Organization; YWHAE: tyrosine 3-monooxygenase/tryptophan 5-monooxygenase activation protein epsilon.

**Table 4:** Clinicopathological and outcome summary—non-AML primary renal mesenchymal tumors (n = 28).

Tumor (n); Age; M–F ratio	Treatment	Key IHC results (current study)	Locoregional recurrence	Metastasis	Status at last contact
Clear cell sarcoma of kidney (CCSK) (n = 7) Pediatric (5): (3, 5, 6, 13, and 13 years); Adult (20 and 34 years) M–F ratio of 6:1	Radical nephrectomy ×7	CCND1 (nuclear)+, all; BCL2 (cytoplasmic)+, all; WT1-; SMA-; synaptophysin-	1 (20 years; abdominal wall; biopsy-confirmed)	3: pN1 retrocaval/ preaortic (3 years) ×1; pN1 interaortocaval (5 years) ×1; renal vein and IVC thrombus with lung metastasis (13 years) ×1	3 Alive; 1 dead (PRMT -related); 3 lost to follow-up
Ewing sarcoma (EWS) (n = 6) Pediatric (2): (14 and 15 years); Adult (4): (20, 31, 38, and 40 years); M–F ratio of 3:3	Radical nephrectomy ×5; nephro-ureterectomy ×1	CD99 membranous+ (5/6); LCA-; CK-; 1 case: synaptophysin+, NSE+, CD56+ (CD99-)	0	0	3 Alive; 3 dead (PRMT-related)
Leiomyo sarcoma (LMS) (n = 6), all adults: (54–83 years) M–F ratio of 4:2	Radical nephrectomy ×3; (Radical nephrectomy + chemotherapy) ×3	SMA+ (5/5); desmin+ (4/5); Pan-CK-; Myogenin-; Ki-67 index: 3–35%	2: Retroperitoneum ×1 (66 years, biopsy-confirmed); renal fossa × 1 (78 years, biopsy-confirmed)	1: Lung metastasis ×1	1 Alive; 5 dead (PRMT-related)
Congenital mesoblastic nephroma (CMN) (n = 2): (a) cellular (3 years); (b) classic (3 months)	Radical nephrectomy ×2 (both)	Vimentin+ (2/2); WT1 focal+ (1/2); BCL2- (helps exclude CCSK)	0	0	1 Alive; 1 lost to follow-up
Malignant rhabdoid tumor (MRT)* (n = 1), 28 years/F	Nephro-ureterectomy + chemotherapy (cyclophosphamide, etoposide, and carboplatin) + radiotherapy	Vimentin+; SMA+; CK focal+; CD99+; S100+; INI1 (SMARCB1)—complete nuclear loss (confirmed; +internal controls);HMB-45-; PAX8-; myogenin-	0	0	Alive
Synovial sarcoma (SS)† (n = 1), 51 years/M (metastatic at diagnosis)	Radical nephrectomy + chemotherapy (ifosfamide, etoposide) + radiotherapy	Vimentin+; CK7 focal+; ALK-; CD10-; Ki-67 index: 30–40%; SS18-SSX IHC done	0	1: Right femoral neck (at presentation)	Dead (PRMT-related)
Mesenchymal chondrosarcoma (MCS) (n = 1), 32 years/M	Radical nephrectomy + chemotherapy + palliative radiotherapy	IHC not performed; recommended: S100+ (cartilaginous nodules), CD99+ and NKX2.2+ (primitive small round cell component); SOX9+ (both components)	0	0	Alive
Inflammatory myofibroblastic tumor (IMT)‡ (n = 1), 40 years/M	*Partial nephrectomy*	*SMA+; Vimentin+; CD68+; ALK+ (ALK1/CD246 clone); CD34-; PAX8-; ZN stain- (excludes infection)*	0	0	Alive
*Angioleiomyoma (n = 1), 56 years/M*	*Partial nephrectomy*	*SMA+; desmin focal+; HMB-45-; CD34-; S100-; Ki-67 index <1% (HMB-45- key discriminator: excludes AML)*	0	0	Alive
*Lipoma§ (n = 1), 1 year/M*	*Incidental Partial nephrectomy (with pyeloplasty)*	*IHC not performed; required: HMB-45, Melan-A, cathepsin K (all must be negative to exclude AML)*	0	0	Alive
*Leiomyoma (n = 1), 9 years/M*	*Partial nephrectomy*	*SMA+; Desmin+; vimentin+; HMB-45-; Pan-CK-; S100-; Ki-67 index < 1%*	0	0	Alive

Notes: One case of classical angiomyolipoma with synchronous epithelial malignancy of the biliary tract showed tumor deposits at station 13 and cystic nodes (metastatic carcinoma confirmed with pan-CK IHC). Epithelioid angiomyolipoma cases with lymph node deposits were not included in metastasis count (see text).

* INI1 (SMARCB1) complete nuclear loss—*sine qua non* for MRT according to WHO 2022 classification; positive in internal controls.

† SS: patient presented with bone metastasis. ^‡^ALK IHC negativity in ~50% of IMT; ALK FISH confirmation recommended.

§ Lipoma: incidental finding during pyeloplasty; melanocytic marker IHC (HMB-45, Melan-A, cathepsin K) not performed—critical limitation.

IVC: inferior vena cava; pN1: pathologically confirmed regional nodal metastasis (CCSK age 3 years—true metastasis); Pan-CK: pan-cytokeratin; IHC: immunohistochemistry; INI1: integrase interactor 1; SMARCB1: SWI/SNF-related, matrix-associated, actin-dependent regulator of chromatin, subfamily B, member 1; ALK: anaplastic lymphoma kinase; WHO: World Health Organization; FISH: fluorescence *in situ* hybridization; HMB-45: human melanoma black-45.

Two EAML cases showed lymph node tumor deposits without features suggestive of aggressive AML, according to Brimo et al. (necrosis, mitosis ≥ 2/10 HPF, atypical mitosis, and ≥70% atypical epithelioid cells) (7); both patients remained disease-free. IHC confirmed the PEComa lineage in two cases. These findings support multicentric PEComa or tumor embolization rather than true metastasis (4, 5). Hence, these AML lymph node deposits were excluded from the metastasis count.

Data for 64 patients were obtained through HIS and phone calls. Follow-up was 41.6 ± 35.9 months (median: 31.8; range: 0.3–139.7), with a cumulative value of 222.1 patient-years. Sixty percent (39/64) had follow-ups of more than 24 months and 27% (17/64) had follow-ups of more than 60 months; 49 (77%) patients were alive; 10 (16%) died due to PRMT-related causes, 1 (2%) died due to unrelated malignancy (epithelial carcinoma of the biliary tract with regional lymph node metastasis concurrent with classical AML), and 4 (6%) were lost to follow-up. All 10 such deaths occurred due to malignant tumors: LMS (5), EWS (3), CCSK (1), and SS (1). No mortality occurred due to benign/intermediate tumors. Lost to follow-up occurred in three patients with CCSK and one patient with CMN (cellular variant).

## Discussion

Renal mesenchymal tumors are an uncommon, heterogenous group arising in the kidney, ranging clinically from high-grade synovial sarcomas to asymptomatic tiny renomedullary interstitial tumors (RMICTs). This series is among the largest from the Indian subcontinent and the first to apply the WHO 2022 biological behavior-based stratification (benign/intermediate/malignant) besides age-based stratification to a South Asian PRMT cohort. The six-category framework—adult benign, adult intermediate, adult malignant, pediatric benign, pediatric intermediate, and pediatric malignant—reflects landmark Western literature series (1, 3) and follows the WHO 2022 taxonomy (2).

The prevalence of PRMTs was slightly lower in this retrospective cohort (3.2%) than 4.4% reported by Alruwaii et al. (3). AML was the most common PRMT (56%), with classical (n = 28), leiomyoma-like (n = 5), and lipoma-like (n = 1) subtypes. Classical and LMAML occurred in adults, peaking in the fourth to sixth decades, with a female predilection; hormonal effects are postulated (15). Differentiation of LLAML from true intrarenal lipoma requires IHC negativity for melanocytic markers (HMB-45, Melan-A, and cathepsin K); even focal positivity reclassifies the lesion as fat-predominant AML (5). IHC was not performed for pediatric lipoma (1 cm), a limitation of this diagnosis. Renal lipoma, despite being a benign lesion, may necessitate germline PTEN testing and genetic counseling in children when it appears alongside other indicators of PTEN hamartoma tumor syndrome (PHTS)/Cowden syndrome (16). We reported an incidentally detected rare case of angioleiomyoma (not recognized as a separate entity according to WHO 2022 classification), which might be a vascular-rich variant of leiomyoma. Surgical excision is the diagnostic and curative criteria. We did not encounter other benign PRMTs, such as hemangioma, juxtaglomerular cell tumor, lymphangioma, schwannoma, and glomus tumor.

According to the WHO 2022 classification, EAML belongs to the category of intermediate-behavior tumors (2), which are fat-poor but mimic RCC on imaging. One EAML had a pediatric occurrence (1/2 cases; 6-year-old girl), indicating a possible TSC association, consistent with another EAML report (15).

The WHO 2022 classification (2) designates CMN subtype with intermediate biological behavior. The classic variant (EGFR-ITD) is benign after complete excision, with a recurrence rate of <2% (9). The cellular variant (*ETV6::NTRK3* gene fusion) is analogous to infantile fibrosarcoma, with a recurrence risk of approximately 10% (9). In cellular CMN, *ETV6::NTRK3* gene fusion serves as a prognostic marker and therapeutic target. Neurotrophic tyrosine receptor kinase (NTRK) inhibitors (larotrectinib and entrectinib) are approved by the US Food and Drug Administration (FDA, USA) and the European Medicines Agency (EMA) for TRK-fusion-positive tumors and as salvage for unresectable or recurrent cellular CMN (11). This highlights the need for FISH or reverse transcription polymerase chain reaction (RT-PCR) testing for *ETV6::NTRK3* fusion in all cellular CMN.

Inflammatory myofibroblastic tumor showed positive ALK IHC. However, 50% of IMT cases are ALK IHC negative, because ALK rearrangements involve fusion partners (RANBP2, CLTC, and TPM3), and some variants cannot be detected by antibody clones (8). For a myofibroblastic spindle cell tumor in a young adult with inflammatory stroma, without infection (Ziehl–Neelsen stain negative [ZN-]) or lymphoma (LCA negative), IMT appears an appropriate diagnosis despite ALK IHC negativity. When clinicopathological features suggest IMT but ALK IHC is negative, escalate to: (i) repeat ALK IHC with an alternative clone (5A4, D5F3, or SP8); (ii) ALK FISH (break-apart probe) to detect rearrangement irrespective of fusion partner; or (iii) identify fusion partner using RNA-based next-generation sequencing (NGS) (8). If confirmed by test, the diagnosis is definitive, and ALK inhibitor therapy is administered. If all modalities are negative, then the diagnoses such as solitary fibrous tumors, LMS, and dedifferentiated liposarcoma must be excluded.

The WHO 2022 classification (2) classifies CCSK as a pediatric renal tumor with a mean age of 3 years at presentation; its occurrence in adults is exceptionally rare (2). Histologically, CCSK exhibits a variety of patterns, including cellular, myxoid, sclerosing, epithelioid, palisading, spindle cell, storiform, and anaplastic, often admixed with classic pattern or with one another (2). In this series, both adult CCSKs showed histopathological morphology and IHC profile (CCND1+, BCL2+, WT1-, synaptophysin-, and SMA-). Cyclin D1 (CCND1) and B-cell lymphoma-2 (BCL2) are practical IHC surrogate markers for CCSK when molecular testing is unavailable. BCOR exon 15 ITD (found in 85–90% of CCSK by PCR, FISH or sequencing) or *YWHAE::NUTM2* oncogenic gene fusion studies are required in the remaining cases (17). Lack of molecular confirmation at the diagnosis is the main diagnostic limitation in two adult patients with CCSK. Patients with stage I disease who underwent lymph node sampling without radiotherapy achieved excellent outcomes, suggesting that radiotherapy can be safely withheld from this subgroup in the treatment protocol. A doxorubicin-containing multiagent chemotherapy protocol (Children’s Oncology Group [COG]) is used with radiotherapy for stage III–IV disease (18). The 20-year-old CCSK patient who developed abdominal wall recurrence followed by disease-related mortality illustrates the aggressive behavior of adult CCSK versus the pediatric form.

Renomedullary interstitial tumors (RMICTs) are benign mesenchymal lesions not identified in our study. These tumors are found in 37% of autopsies (4) but in only 0.2% of nephrectomy series (19), possibly because they are asymptomatic, small in size (~1–7 mm), and located in the medulla. In contrast to the 17% of cases reported by Alruwaii et al. (3), the data on RMICT are sparse in the Indian literature; an Indian allograft biopsy case and one autopsy are the only reports (20, 21).

In this heterogeneous tumor cohort, follow-up of 41.6 ± 35.9 months was assessed to evaluate short-to-intermediate-term outcomes. Of the 11 deaths, 10 (91%) were attributable to PRMT, while 1 (9%) was due to an unrelated malignancy (biliary tract carcinoma) in an AML patient, which behaved benignly. PRMT-related mortality occurred only in the malignant cohort, predominantly LMS and EWS, supporting the WHO 2022 biological behavior classification (2). Although the follow-up loss was 6%, late relapses in tumors, such as CCSK, cannot be excluded.

The present findings align with the published literature. The prevalence of PRMT and the predominance of AML are similar to those in prior surgical series (1, 3). Nonetheless, AML is rare in the pediatric population, where its occurrence may suggest an association with TSC. Among the tumors with intermediate behavior, we did not encounter any cases of solitary fibrous tumor. The frequency of malignant tumors reflects referral bias at a tertiary oncology center. LMS and EWS were the most common types in adults. Interestingly, both CCSK and MRT were observed in adult patients, underscoring their broader age range and clinical significance, even though they typically affect younger individuals. Follow-up was consistent with previous institutional data. PRMT-related mortality was limited to malignant tumors (1, 3). The study highlights the significance of understanding rare subtypes, precise histopathological diagnosis, multidisciplinary treatment approach, and careful long-term monitoring to improve outcomes, particularly for tumors exhibiting aggressive or ambiguous behavior. These findings emphasize the need for integrating morphology, IHC, and selective molecular testing in rare diagnostically challenging cases to guide management.

Malignant soft tissue tumors, which are reported in the literature but not encountered in our study, include angiosarcoma, rhabdomyosarcoma, osteosarcoma, undifferentiated pleomorphic sarcomas, and desmoplastic small round cell tumors.

Mesenchymal kidney tumors in children are much rarer and less varied. CMN is a common benign infantile tumor. We encountered its two cases, both presenting as incidental renal masses. The ossifying renal tumor in infancy is another entity reported in the literature but was not found in our survey (2).

Study limitations include a retrospective single-center design, small numbers of several rare tumor subtypes, and limited molecular testing. Multicenter studies with larger cohorts are needed for validation.

Finally, coming to the context and strengths of this series, most PRMT series are autopsy-based or include dominant and incidental tumors with synchronous pathology, unlike the current nephrectomy-based series. The current study provides data from a North Indian referral center using the WHO 2022 classification (2), IHC, and outcome stratification, where resource constraints limit molecular testing. These data reflect population-level diagnostic challenges for South Asian pathologists that affect management of PRMT. Rare entities, such as primary renal angioleiomyoma, adult MRT with INI1 loss on IHC, and adult CCSK in a single-institution decade-long series, illustrate diagnostic difficulties even in predominantly AML-dominated PRMT cohorts. Table 5 summarizes the differential diagnoses, IHC profile, and molecular tests, aiding the diagnosis of PRMTs and the therapeutic implications of the studied tumor entities.

**Table 5:** Primary renal mesenchymal tumors—IHC profile, differential diagnosis, molecular testing, and treatment.

Tumor (n)		Key histopathological features	Key diagnostic IHC markers	Important differential diagnosis (key discriminator)	Molecular test (if indicated)	Standard treatment
**Benign tumors**					
Classical AML (n = 28)	Adipose tissue, dysmorphic thick-walled vessels without elastic lamina, myoid spindle cells	HMB45+, Melan-A+, cathepsin K+ (100% sensitive in all AML subtypes) (22), SMA+	• RCC, clear cell type: sarcomatoid differentiation or fat invasion (PAX8+, CK+) (2, 5) • Leiomyoma: HMB-45-, Melan-A- (2, 5) • Leiomyosarcoma—if fat-poor AML (HMB-45-, Melan-A-) (5) • Well-differentiated liposarcoma (MDM2/CDK4+)—if fat-predominant	• TSC1/TSC2 deletion (NGS/FISH) if TSC suspected (23)	• <4 cm, asymptomatic: surveillance • Symptomatic/≥ 4 cm: embolization or ablation • Large/complex: surgical excision • TSC-associated/ unresectable: everolimus /sirolimus (24)
Leiomyoma-like AML (n = 5)	Smooth muscle predominant AML	HMB45+, cathepsin K+, SMA+	• Leiomyoma (HMB-45-, SMA+, desmin+) (25) • Angioleiomyoma (HMB-45-; CD34+ vessels) (6) • Leiomyosarcoma (high-grade histology: mitoses/ nuclear atypia/pleomorphism/ hyperchromasia+; SMA+, HMB-45- ) (26) • Dedifferentiated liposarcoma: (MDM2/CDK4+; no melanocytic markers) (27)	• MDM2 FISH exclude dedifferentiated liposarcoma (27)	• Surgical excision (nephron-sparing preferred) • No adjuvant therapy • TSC surveillance (if TSC-associated) (24)
Lipoma-like AML (n = 1)	Mature adipose tissue with focal PEComa cells	Focal HMB45+, Melan-A+, cathepsin K+	• True intrarenal lipoma (HMB45-, Melan-A-) (2, 5) • Well-differentiated liposarcoma **(**MDM2/CDK4+) (27)	• MDM2 FISH exclude well-differentiated liposarcoma (27) • *PTEN* sequencing if true lipoma in child: screen for Cowden syndrome (16)	• Surgical excision TSC surveillance if AML confirmed (24) • Genetic counseling if true lipoma in child (16)
Angioleiomyoma (n = 1; exceptionally rare with <10 cases globally)	Thick vessels with smooth muscle bundles	SMA+, desmin+, HMB45-, Ki-67 <1% ≥excludes LMS; HMB45-/ cathepsin K-/ Melan-A-(all 3 melanocytic markers must be negative to exclude AML) (5, 22)	• Leiomyoma-like AML: (HMB-45+) (5, 22) • True renal leiomyoma: no thick-walled vessels (SMA+, HMB-45-) (25) • Leiomyosarcoma: high-grade histology; SMA+, HMB-45-(see LMS row below) (26) • Solitary fibrous tumor (STAT6+, CD34+) (1)	None	• Surgical excision (partial nephrectomy; curative) • Excellent prognosis • No adjuvant therapy required
*Lipoma (n = 1)*	Mature adipocytes	*HMB45-*, *Melan-A-*	*• Lipoma-like AML even focal HMB-45/Melan-A/cathepsin K+ ≥AML; see above* (5, 22) *• Well-differentiated liposarcoma (MDM2/CDK4+)* (27)	• MDM2 FISH (27) • *PTEN* sequencing if true lipoma in child: screen for Cowden syndrome (16)	*• Observation if incidental*, *small*, *benign* *• Surgical excision if symptomatic or growing* *• Genetic counseling for Cowden syndrome (*PTEN*) if true lipoma in child* (16) *• No adjuvant therapy*
*Leiomyoma (n = 1)*	Bland spindle fascicles	*SMA+*, *Desmin+*, *HMB45-*	*• Leiomyoma-like AML HMB-45+/cathepsin K+ (see AML rows above)* (5, 22, 26) *• Angioleiomyoma thick-walled vessels; HMB-45-; see above* (6) *• Leiomyosarcoma: high-grade histology; see LMS row below* (26)	*No specific molecular testing required* *• MDM2 FISH if* *dedifferentiated liposarcoma is a differential* (27)	*• Surgical excision nephron-sparing if feasible; curative* *• No adjuvant therapy* *• Excellent prognosis* *• Surveillance annual imaging for 1–2 years if large* (25)
**Intermediate-behavior tumors**					
*EAML* * (n = 2)	Epithelioid PEComa	HMB45+, Melan-A+, cathepsin K+	• Clear cell RCC (PAX8+, CK+, CAIX+) (2) • Translocation RCC (Xp11.2) (PAX8+, TFE3+) (2) • Chromophobe RCC (PAX8+, CD117+, CK7+) • Oncocytoma (PAX8+, CD117+, CK7-) • Malignant melanoma (S100+, SOX10+, Melan A+, HMB45+, cathepsin K-, SMA-, cytokeratin-) • Epithelioid angiosarcoma (CD31+/ERG+) (2)	• TFE3/TFEB FISH (MiT-family translocation RCC) (2) • MDM2 FISH if dedifferentiated liposarcoma is a differential (27)	• Surgical excision • mTOR inhibitors (everolimus/sirolimus) for TSC-associated or unresectable EAML (24) • LN deposits without malignant criteria: surveillance only (7)
*IMT* *† (n = 1)*	Myofibroblastic spindle cells with inflammation	*ALK+*, *SMA+*	*• Leiomyosarcoma (ALK-)* (8) *• Fibrosarcoma ≥diagnosis of exclusión* (8) *• Solitary fibrous tumor (STAT6+)* (1) *• Dedifferentiated liposarcoma* ***(****MDM2/CDK4+)* (27) *• Xanthogranulomatous pyelonephritis (abundant CD68+ foamy histiocytes*, *lacks true spindle-cell neoplasm, ALK-)* (8)	*• ALK FISH (break-apart)—confirms rearrangement; identifies ALK inhibitor eligibility* *• RNA-NGS fusion panel if ALK IHC negative (RANBP2, CLTC*, *TPM3 fusions)* (8) *• MDM2 FISH if dedifferentiated liposarcoma considered* (27)	*• Surgical excision* *• ALK-rearranged unresectable/recurrent: crizotinib/lorlatinib* (8) *• ALK-negative: excision + surveillance; FISH recommended* (8)
*CMN (n = 2; classic = 1, loose stroma, low mitosis; cellular = 1*, *hypercellular, sinus invasion, higher mitosis)*	Infantile spindle tumor	*Vimentin+*, *cyclin D1+ (cellular variant)* (9, 10)*, WT1 focal+*	• Metanephric stromal tumor ≥alternating, nodular cellularity, angiodysplasia, onion skinning (CD34+, BRAF V600E+) (2) • Stromal type Wilms tumor (strong WT1+) (10) • CCSK (BCOR+, BCL2+) (17) • IMT (ALK+) (8)	• ETV6::NTRK3 FISH/ RT-PCR—cellular variant (required; therapeutic target—NTRK inhibitors) (9–11) • EGFR-ITD sequencing—classic variant (9) • BRAF V600E mutation detection (PCR, NGS)	• Surgical excision (curative in classic variant) (9, 10) • Cellular + sinus invasion: close surveillance (10) • Recurrent ETV6::NTRK3+ CMN: larotrectinib/ entrectinib (FDA/EMA approved) (11)
***Malignant tumors***					
*CCSK* *‡ (n = 7)*	Delicate branching vasculature, myxoid stroma	*BCOR+*, *cyclin D1+, BCL2+*	*• Wilms tumor (blastemal; strong WT1+)* (17) *• Cellular CMN (cyclin D1+, BCL2-)* (9, 17) *• Clear cell RCC 9 (PAX8+, CK+, CAIX+)* (2) *• MRT (INI1 loss)* (2) *• Extraskeletal EWS (CD99+, NKX2.2+)* (2)	*• BCOR exon 15 ITD by PCR, FISH/ sequencing—85–90% of CCSK; mandatory in adult cases‡* (17, 18) *• YWHAE::NUTM2* *FISH/sequencing if BCOR negative (~5–10%)* (17) *• NGS panel if FISH unavailable* (1, 2)	*• Surgical excision* *• Doxorubicin-based multiagent chemotherapy (COG protocol)* (18) *• Radiotherapy—tumor bed; mandatory for stages III and IV* (18)
*EWS* *(n = 6) PNET* *terminology abolished (WHO 2022)* (2)	Small round blue cells (sheets and rosettes)	*Diffused CD99+, NKX2.2+*	*• Wilms tumor (blastemal predominant; strong WT1+, N-terminus)* (28) *• Lymphoma (CD45+)* (28) *• Rhabdomyosarcoma (CD99+*, *desmin+*, *myogenin+)* (28) *• Poorly differentiated synovial sarcoma (EMA+, CK+, TLE1+, SS18::SSX+)* (1) *• Small cell carcinoma (CK+, INSM1+*, *synaptophysin+*, *chromogranin+)* (28) *• CIC-rearranged sarcoma (ETV4+*, *WT1+*, *CD99 patchy, NKX2.2-)* (28)	*• EWSR1 FISH (break-apart)—required before chemotherapy* *• RT-PCR: EWSR1::FLI1 (~85%)* *• RT-PCR: EWSR1::ERG (~10%)* *• NGS if EWSR1 negative* (28)	*• VDC/IE chemotherapy (vincristine, doxorubicin, cyclophosphamide/ ifosfamide*, *etoposide)* *• Surgical excision* *• Local radiotherapy—unresectable or margin+* (28)
LMS (n = 6)	Malignant spindle smooth muscle tumor; high-grade histology (increased mitoses/nuclear atypia+/ pleomorphism+/ hyperchromasia+/ LVI±)	*SMA+*, *desmin+*, *h-caldesmon+*	• Sarcomatoid RCC—most critical (PAX8+, CK+) (1, 2) • IMT (ALK+) (8) • EAML (melanocytic markers+) (29) • Fibrosarcoma ≥diagnosis of exclusion (29) • Synovial sarcoma (EMA+, TLE1+, SS18::SSX+) (29) • Malignant peripheral nerve sheath tumor (MPNST; (SOX10+, S100+) (29) • Dedifferentiated liposarcoma (MDM2/CDK4+) (29)	• MDM2/CDK4 FISH exclude dedifferentiated liposarcoma (27) • SMARCB1 (INI1) IHC must be retained in LMS (2) • SS18::SSX FISH exclude leiomyosarcoma	• Surgical excision • Metastatic/inoperable: doxorubicin ± ifosfamide • 2nd line: trabectedin/ pazopanib (30)
MRT§ (n = 1)	Rhabdoid cells	*INI1 loss*	*• Sarcomatoid RCC (rhabdoid features; PAX8+, CK+)* (2) *• SMARCA4-deficient renal carcinoma* ***(****SMARCA4/BRG1 loss; INI1 retained)* (2) *• Rhabdomyosarcoma (myogenin+*, *MyoD1+)* (2) *• Undifferentiated pleomorphic sarcoma (INI1 retained; no rhabdoid inclusions)* (2)	*• SMARCB1 sequencing—biallelic inactivation (deletion/ mutation)* (2, 31) *• SMARCA4 IHC helps exclude SMARCA4-deficient carcinoma* (2)	*• Surgical excision* *• Multiagent chemotherapy (doxorubicin*, *cyclophosphamide ± carboplatin/etoposide)* (30) *• EZH2 inhibitor (tazemetostat)—clinical trials; FDA-approved for epithelioid sarcoma* (32)
SS (n = 1)	Monophasic spindle tumor	*TLE1+, SS18-* *SSX IHC+*	*• Sarcomatoid RCC (PAX8+, CK+)* (1) *• Solitary fibrous tumor (STAT6+*, *CD34+)* (1) *• IMT (ALK+)* (8) *• Fibrosarcoma ≥diagnosis of exclusión* (33)	*• SS18::SSX FISH— pathognomonic; mandatory for confirmation* *• TLE1 IHC—first-line screen (~95% sensitive)* *• RT-PCR: SS18::SSX1/2/4 if FISH unavailable* (1, 33)	*• Localized: surgical excision + radiotherapy* *• Metastatic: doxorubicin ± ifosfamide* *• 2nd line: trabectedin/ pazopanib* (30)
MCS (n = 1)	Biphasic cartilage + primitive cells	*SOX9+*, *S100+*, *CD99+*	• Ewing sarcoma—most critical (CD99+, NKX2-2+) (2, 28) • Dedifferentiated chondrosarcoma (MDM2/ CDK4+) (2) • Small cell carcinoma (CK+, INSM1+, synaptophysin+, chromogranin+) (2) • Synovial sarcoma (biphasic)—epithelial component; SS18::SSX fusion; no cartilage (33) • CCSK—fibromyxoid stroma; CCND1+; no cartilage (17)	• HEY1::NCOA2 FISH (~70% of MCS— confirmatory) (2) • IRF2BP2::NCOA2 FISH (if HEY1 negative) (2) • IDH1/IDH2 sequencing (exclude dedifferentiated chondrosarcoma) (2)	• Surgical excision (complete resection—key outcome determinant) • Adjuvant chemotherapy: VDC/ IE protocol • Radiotherapy: margin+ or unresectable (34)

AML: angiomyolipoma; EAML: epithelioid AML; LMAML: leiomyoma-like AML; LLAML: lipoma-like AML; IMT: inflammatory myofibroblastic tumor; CMN: congenital mesoblastic nephroma; CCSK: clear-cell sarcoma of the kidney; EWS: Ewing sarcoma; LMS: leiomyosarcoma; MRT: malignant rhabdoid tumor; SS: synovial sarcoma; MCS: mesenchymal chondrosarcoma; RCC: renal cell carcinoma; SFT: solitary fibrous tumor; SMA: smooth muscle actin; CK: keratin; LCA: leucocyte common antigen (CD45); FISH: fluorescence *in situ* hybridization; NGS: next-generation sequencing; IHC: immunohistochemistry; TSC: tuberous sclerosis complex; PNET: primitive neuroectodermal tumor (obsolete according to WHO 2022).

* EAML lymph node deposits in both cases: malignant histological criteria according to Brimo et al. (7) were not met; all patients alive without progression—interpreted as multifocal PEComa, not true metastasis.

† ALK IHC positive in the present IMT case (ALK1/CD246 clone). In ~50% of IMT, ALK IHC is negative due to non-detectable fusion partners; escalation pathway: (1) repeat IHC with 5A4, D5F3 or SP8 clone; (2) ALK FISH break-apart; (3) RNA-NGS fusion panel. ALK rearrangement confirmation identifies eligibility for ALK inhibitor therapy (8).

‡ Adult CCSK (age: 20 and 34 years) constitutes an exceptional diagnostic claim according to WHO 2022 (2, 14); BCOR exon 15 ITD FISH or YWHAE::NUTM2 FISH is mandatory for molecular confirmation (14, 15).

§ INI1 (SMARCB1) complete nuclear loss with retained staining in internal controls (lymphocytes and endothelial cells) was confirmed—this is the diagnostic *sine qua non* for MRT according to WHO 2022; positive internal controls are necessary for result validity (2, 28).

## Conclusions

This study is the largest Indian nephrectomy-based PRMT series applying the WHO 2022 classification (2). Comprehensive histopathology with focused IHC remains the mainstay of tumor diagnosis, while selective molecular tests improve classification and treatment-related choices in diagnostically challenging tumors. Prospective multicenter studies with extensive molecular profiling are necessary.

## Data Availability

All data generated or analyzed in this study are included in this article.
